# Bilateral Adrenal Hemorrhage and Adrenal Insufficiency in the Context of Polycythemia Vera: A Case Report and Review of the Literature

**DOI:** 10.1155/2022/5335543

**Published:** 2022-06-22

**Authors:** Sara Soltanmohammadi, Mehrbod Vakhshoori, Ghazaleh Sajadi, Maryam Heidarpour

**Affiliations:** ^1^Isfahan Endocrine and Metabolism Research Center, Isfahan University of Medical Sciences, Isfahan, Iran; ^2^Heart Failure Research Center, Cardiovascular Research Institute, Isfahan University of Medical Sciences, Isfahan, Iran

## Abstract

**Background:**

Polycythemia vera (PV) is a myeloproliferative disorder presented with different manifestations. However, bilateral adrenal hemorrhage (BAH) and adrenal insufficiency (AI) are rare manifestations. Herein, we described a patient who suffered from BAH and AI in the context of PV. *Case Presentation*. A 60-year-old man with an underlying history of PV was admitted with severe abdominal pain, nausea, and loss of consciousness. Primitive computed tomography (CT) scan findings revealed bilateral adrenal masses (right: 40 *∗* 23 mm, left: 60 *∗* 35 mm) with the second scan showing quite similar results (right adrenal: 40 *∗* 29 mm, left adrenal: 48 *∗* 26 mm) suggesting BAH. The further adrenal assessment proved concurrent AI. Both adrenal masses resolved completely after 15 months. However, the patient still suffered from AI.

**Conclusion:**

BAH and AI in patients with PV should be considered rare complications requiring high clinical suspicion for early diagnosis and treatment to avoid life-threatening outcomes.

## 1. Background

Adrenal hemorrhage (AH) is a rare disorder with a prevalence ranging from 0.14 to 1.8% reported from post-mortem records [1, 2]. Several predisposing factors have been suggested for this unusual disease, including septic shock, stress, abdominal trauma, pregnancy, coagulopathies, and different hematologic disorders such as antiphospholipid syndrome and essential thrombocytosis [2, 3]. However, the incidence of bilateral adrenal hemorrhage (BAH) and subsequent adrenal insufficiency (AI) in the context of polycythemia vera (PV), as one of the myeloproliferative disorders, is rarely reported [4, 5].

Herein, we described a patient with an underlying history of PV who experienced BAH and subsequent AI and reviewed the literature in this regard.

## 2. Case Presentation

A 60-year-old man with underlying hypertension and PV was admitted after an acute onset of severe abdominal pain, nausea, and concomitant loss of consciousness. On admission, he had a blood pressure of 110/75 mmHg. The patient mentioned prior episodes of vague abdominal pain over the past decade. He was diagnosed with PV 14 years ago on the basis of erythrocytosis, leukocytosis, and a bone marrow biopsy which was consistent with erythroid and myeloid hyperplasia without any grades of fibrosis. A PCR test for BCR-ABL1 was negative and the mutation JAK2V617F was detected. He denied using cigarettes and alcohol. His only medications were daily aspirin (80 mg), hydroxyurea (200 mg), and irregular usage of losartan (25 mg) and he did not mention usage of any other drugs. He irregularly performed phlebotomies and did not have good adherence to treatment. Based on previous medical records, his underlying disease was not well controlled and his last cell blood count revealed white blood cells (WBC) of 19.2 *∗* 10^3^/l, hemoglobin of 14.2 g/dL, and platelet counts of 160 *∗* 10^6^/l. There were no records indicating a hypertension control status. He denied any previous thrombotic events or any major bleeding episodes. On physical examination, he was lethargic and confused. Abdominal tenderness was present in the epigastrium as well as the periumbilical region, and splenomegaly was also noted in addition to left side costovertebral angle tenderness. As shown in Table 1, laboratory data were normal except for leukocytosis, which had been approved to be in the same range since he was diagnosed with PV.

Abdominopelvic spiral computed tomography (CT) scan with intravenous and oral contrast was requested and revealed splenomegaly and a hypodense lesion with a mean 55 Hounsfield unit (HU) at the site of the right adrenal gland (40 *∗* 23 mm) and another ill-defined soft tissue lesion (60 *∗* 35 mm) with infiltrative margins at the site of the left adrenal gland without any evidence of vascular thrombosis. Supportive therapy was initiated, but the patient left the hospital due to personal desire and was admitted to one of the tertiary hospitals in Isfahan, Iran.

His vital signs on admission were the followings: blood pressure: 115/85 mmHg, heart rates: 100/min, respiratory rates: 20/min, T: 37.1°C, O_2_ saturation: 85% on room air (94% with a face mask). He suffered from dyspnea, chest pain, and diarrhea. On physical examination, huge splenomegaly, generalized abdominal tenderness, and left costovertebral angle tenderness were noted. The requested electrocardiogram (ECG) showed sinus tachycardia. Laboratory profile showed elevated creatinine, lactate dehydrogenase (LDH), and troponin (Table 1).

**Figure 1:**
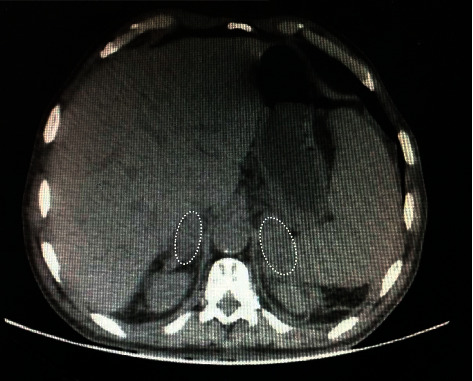
Bilateral adrenal masses in favor of adrenal hematoma.

With prior suspicion of prerenal azotemia due to intraabdominal disorders including mesenteric ischemia, fluid replacement and broad-spectrum antibiotics were prescribed. Due to the presence of multiple air-fluid levels on the abdominal X-ray, surgical consultation was requested, which recommended observation and correction of fluid and electrolyte imbalances. After two days, the patient's symptoms improved, and he gained normal bowel function with normal creatinine. Due to the suspicion of adrenal disorders including probable pheochromocytoma or adrenal cancer, an abdominal CT scan with the adrenal protocol was performed that revealed huge splenomegaly (spleen span: 25 cm) and bilateral adrenal masses (right adrenal gland: 40 *∗* 29 mm, left adrenal gland: 48 *∗* 26 mm) without any enhancement and 50 HU subsequently in favor of adrenal hematoma (Figure 1). Comprehensive adrenal glands assessment was performed and revealed the followings: aldosterone; 45 pg/ml (reference range: 30–400 pg/ml), plasma renin; 12 pg/ml (reference range: 12–36 pg/ml), 17 (OH) progesterone; 1.3 ng/ml (reference range: 0.2–2.3 ng/ml) and dehydroepiandrosterone sulfate (DHEAS); 25 mcg/dl (reference range: 56–80 mcg/dl). A 24-hour urine analysis was also requested, and the following were reported: norepinephrine; 23.1 mcg/day, epinephrine; 0.7 mcg/day, normetanephrine; 112 mcg/day, metanephrine; 62 mcg/day, and vanillylmandelic acid; 6 mg/day. These finding collections including nausea, vomiting, and abdominal pain, and abnormal adrenal CT findings were in respect to nonfunctional adrenal masses that resulted in AI in the context of adrenal hematoma. Therefore, basal cortisol was checked and the result was less than 3 mcg/dl. A tetracosactide test was performed in a way that one milligram of tetracosactide was injected intramuscularly and the cortisol level was measured at baseline and 4 hours afterwards (basal serum cortisol at 8 AM; 2.28 mcg/dl, 4 hours; 10.37 mcg/dl). Finally, the AI was confirmed and the patient was treated with daily prednisolone (5 mg) and fludrocortisone (100 microgram). The patient was discharged in a stable condition after 12 days with a recommendation for a follow-up ultrasound.

**Figure 2:**
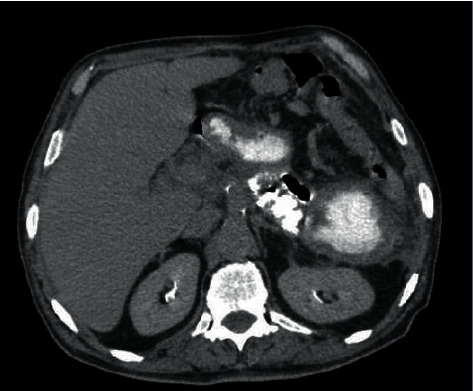
Resolved bilateral adrenal hematoma during follow-up assessment.

During the follow-up assessment, the patient acknowledged voluntarily discontinuing medication for AI after ten months of discharge. However, he had no clinical symptoms of AI. The adrenal hematoma was resolved gradually through periodic ultrasound assessments, and there was no evidence of adrenal masses on the abdominal CT scan 15 months after his discharge (Figure 2). Basal serum cortisol was 5.90 mcg/dL, and serum adrenocorticotropic hormone (ACTH) was 68.2 pg/ml. Further investigation with the tetracosactide test 4 hours after intramuscular injection of 1 mg tetracosactide revealed a serum cortisol level of 18.15 mcg/dL. Therefore, AI was documented, and daily prednisolone (5 mg) was prescribed again.

## 3. Discussion and Conclusions

We reported an Iranian male patient with an underlying condition of myeloproliferative neoplasia (MPN), most likely a PV, which manifested with signs and symptoms of BAH and resultant AI. Although this entity is quite rare in literature, high clinical suspicion is required for prompt diagnosis and treatment. PV is defined as MPN with pathologic growth-factor independent increased growth of erythroid precursors. In addition, myeloid and megakaryocytic lineages are also often affected, resulting in increased numbers of WBCs and platelets. JAK2V617F is the main culprit in this regard [6]. Most common symptoms of PV are attributed to hyper-viscosity leading to oxygen delivery impairment and headache, vision changes, fatigue, claudication, dizziness, and aquagenic pruritus, among commonly reported complaints [6]. To the best of our knowledge, few reports described MPN/PV patients with concomitant BAH and/or AI (Table 2). Gonen et al. reported a 56-year-old male admitted with nausea, vomiting, epigastric pain, and loss of appetite. His lab data revealed erythrocytosis and leukocytosis with confirmed PV diagnosis through bone marrow biopsy. A CT scan revealed bilateral adrenal masses measured 4 *∗* 5 *∗* 5.5 cm and 4 *∗* 3 *∗* 2 cm in the right and left adrenal glands, respectively. Basal serum cortisol of <138 nmol/l, ACTH of 550 pmol/l, and an ACTH stimulation test suggested simultaneous AI. During the periodic follow-up assessment, both adrenal lesions diminished significantly, and they were completely disappeared after two years [5]. Another report was of an 85-year-old man who suffered from PV presented with abdominal tenderness and syncope finally diagnosed as BAH [7]. Bhandari et al. described a 64-year-old man with a chronic history of PV admitted with fever, tachycardia, hypotension, and hypoglycemia. Further assessments were in favor of cardiogenic shock resulting from myocardial infarction. After percutaneous coronary intervention and stent insertion in the left anterior descending artery, he was still hypotensive and low random cortisol (3.3 mcg/dl) with a dramatic response to hydrocortisone stress dose proved AI as the main culprit. Also, a noncontrast CT scan revealed BAH (right adrenal: 5.3 *∗* 3.4 *∗* 3.8 cm, left adrenal: 6.1 *∗* 4.3 *∗* 5.4 cm) [8].

Despite the quite low prevalence of AH, improvement in imaging technologies including sonography, magnetic resonance imaging (MRI) and CT scan might yield to diagnose this topic more frequently with the latter being the modality of choice in adults [9]. Adrenal hematomas appear round or oval, and peri-adrenal fat stranding is almost present. Acute or subacute adrenal hematomas contain high attenuation areas that usually range from 50–90 HU. Most adrenal hematomas resolve gradually over time, but some of them may resolve partially and cause permanent AI and lifelong hormone replacement requirements [4, 8, 10].

The exact pathophysiological mechanisms of AH remain uncertain; however, the anatomic location and physiological properties might play roles. Three main arteries (the superior, middle, and inferior suprarenal arteries) in comparison to a single adrenal vein draining blood from adrenal glands might predispose to vascular congestion and subsequent AH. On the other hand, catecholamine release leads to vasoconstriction of the adrenal veins impairing blood outflow. Efferent adrenal vein thrombosis and hyperviscosity resulting from PV impair venous drainage and venous congestion, ultimately predisposing to AH [1, 8].

In conclusion, BAH with subsequent AI should be considered in the differential diagnosis of patients with myeloproliferative diseases, especially PV, presented with non-specific symptoms including nausea, vomiting, abdominal pain, and hypotension, in order to prevent life-threatening outcomes.

## Figures and Tables

**Table 1:** Laboratory results of the admitted patient^*∗*^.

Laboratory data	First result	Second result	Reference ranges
White blood cells (^*∗*^10^3^/l)	28.5	24.2	4–11
PMN (%)	92	93	45–77
Lymph (%)	5	5	14–44
Mix (%)	3	2	4–20
Hemoglobin (g/dl)	15.5	15.6	14–17.5
Hematocrit	50%	50%	41.5–50.5
MCV (fL)	83.1	83	80–96
MCH (pg)	25.5	25.5	27–33
MCHC (g/dL)	30.7	30.7	31.5–35
Platelets (^*∗*^10^6^/l)	313	198	150–400
PTT (sec)	49	48	25–36
PT (sec)	14	—	10–13
INR	1.1	1.29	1–1.2
Blood urea nitrogen (mg/dl)	13.2	40	8.4–27.5
Creatinine (mg/dl)	1.07	4	0.86–1.4
Sodium (mEq/l)	138	138	135–145
Potassium (mEq/l)	3.8	4.9	3.6–5.2
Blood sugar (mg/dl)	206	74	60–99
Amylase (U/l)	59	33	10–100
Lipase (U/l)	13.8	14	<60
Aspartate aminotransferase (U/l)	16	20	10–40
Alanine aminotransferase (U/l)	16	16	10–41
Alkaline phosphatase (U/l)	309	221	80–306
Total bilirubin (mg/dl)	0.8	0.8	0.1–1.1
Direct bilirubin (mg/dl)	0.47	0.3	0.1–0.3
LDH (U/l)	625	680	140–500
Troponin I (ng/l)	Negative	147.2	<0.1
Calcium (mg/dl)	—	8.5	8.6–10.3
Phosphorus (mg/dl)	—	5	2.8–4.5
Albumin (g/dl)	—	3.5	3.4–5.2
Magnesium (mg/dl)	—	2.1	1.3–2.1
CPK (U/l)	—	86	30–200
ESR (mm)	—	3	Male: 0–22 Female: 0–29
CRP (mg/dl)	—	75	0–6
Uric acid (mg/dl)	—	12.4	3.5–7.2
Venous blood gas			
PH	—	7.30	7.33–7.43
PCO_2_	—	32	41–51
HCO_3_	—	17	24–28
Urine analysis			
Specific gravity	—	1025	
Protein	—	+	
Red blood cell	—	Many	
White blood cell	—	6–8	
Bacteria	—	Few	
Fluid cultures			
Urine culture	—	Negative	
Blood culture (two times)	—	Negative	

^*∗*^The reticulocyte count and peripheral blood smear were not assessed. PMN: polymorphonuclear, MCV: mean corpuscular volume, MCH: mean corpuscular hemoglobin, MCHC: mean corpuscular hemoglobin concentration, PTT: partial thromboplastin time, PT: prothrombin time, INR: international randomized ratio, LDH: lactate dehydrogenase, CPK: creatine phosphokinase, ESR: erythrocyte sedimentation rate, and CRP: C-reactive protein.

**Table 2:** Summary of studies reported adrenal hemorrhage in the context of polycythemia vera.

Authors	Sex	Age (years)	Presenting symptoms	Laboratory data and physical findings	CT findings	Patient management	Patient outcome
Bhandari et al. [8]	Male	64	(i) Unresponsiveness	(i) Hypotension (BP: 72/43 mmHg) (ii) Tachycardia (iii) Fever (T: 38.8°C) (iv) Hypoglycemia (blood sugar: 38 mg/dl) (v) Leukocytosis (vi) Acute kidney injury (vii) Hyperkalemia (viii) Elevated troponin (ix) Elevated coagulation indices (x) Random cortisol: 3.3 mcg/dl	Bilateral adrenal hemorrhage: (i) Right adrenal: 5.3 *∗* 3.4 *∗* 3.8 cm (ii) Left adrenal: 6.1 *∗* 4.3 *∗* 5.4 cm	Stress dose of hydrocortisone (100 mg)	Undertreatment of primary adrenal insufficiency
Gonen et al. [5]	Male	56	(i) Nausea (ii) Vomiting (iii) Epigastric pain (iv) Loss of appetite	(i) Hematocrit: 62% (ii) Leukocytosis (iii) Blood glucose: 65 mg/dl (iv) Basal plasma cortisol: <138 nmol/l (v) ACTH: 550 pmol/l (vi) Splenomegaly	Bilateral adrenal masses: (i) Right adrenal: 4 *∗* 5 *∗* 5.5 cm (ii) Left adrenal: 4 *∗* 3 *∗* 2 cm	Prednisolone	Fully disappearance of adrenal lesions after 2 years
Gelfand et al. [7]	Male	85	(i) Abdominal tenderness (ii) Syncope (iii) Severe chest and abdominal pain	(i) Platelet: 538 *∗* 10^9^/l (ii) Hematocrit: 0.45 (iii) Red cells: 5.7 *∗* 10/l (iv) White blood cells: 15 *∗* 10/l (v) Normal cortisol	Bilateral high-attenuating homogenous adrenal masses	NA	NA

CT: computed tomography, BP: blood pressure, ACTH: adrenocorticotropic hormone, and NA: not available.

## Data Availability

The datasets generated during and/or analyzed during the current study are not publicly available due to confidential issues but are available from the corresponding author upon reasonable request.

## Abbreviations

AH: Adrenal hemorrhage
BAH: Bilateral adrenal hemorrhage
AI: Adrenal insufficiency
PV: Polycythemia vera
WBC: White blood cell
CT: Computed tomography
HU: Hounsfield unit
ECG: Electrocardiogram
LDH: Lactate dehydrogenase
DHEAS: Dehydroepiandrosterone sulfate
ACTH: Adrenocorticotropic hormone
MPN: Myeloproliferative neoplasia
MRI: Magnetic resonance imaging.
